# The Role of the Dietitian within Family Therapy for Anorexia Nervosa (FT-AN): A Reflexive Thematic Analysis of Child and Adolescent Eating Disorder Clinician Perspectives

**DOI:** 10.3390/nu16050670

**Published:** 2024-02-27

**Authors:** Cliona Brennan, Julian Baudinet, Mima Simic, Ivan Eisler

**Affiliations:** 1 Maudsley Centre for Child and Adolescent Eating Disorders, South London, and Maudsley NHS Foundation Trust, De Crespigny Park, Denmark Hill, London SE5 8AZ, UK; julian.baudinet@kcl.ac.uk (J.B.); mima.simic@slam.nhs.uk (M.S.); ivan.eisler@kcl.ac.uk (I.E.); 2 Institute of Psychiatry, Psychology and Neuroscience, King’s College London, De Crespigny Park, London SE5 8AZ, UK; 3 Department of Human Nutrition and Dietetics, School of Health Sciences, London Metropolitan University, 166-220 Holloway Road, London N7 8DB, UK; 4 Department of Child and Adolescent Psychiatry, Institute of Psychiatry, Psychology and Neuroscience, King’s College London, De Crespigny Park, London SE5 8AZ, UK

**Keywords:** anorexia nervosa, adolescents, dietetics, family therapy for anorexia nervosa, reflexive thematic analysis

## Abstract

Background: Despite dietitians being important members of the multidisciplinary team delivering family therapy for anorexia nervosa (FT-AN), their specific responsibilities and roles are unclear and their involvement in the treatment can be a contentious issue. Methodology: Clinicians (*n* = 20) experienced in the delivery of FT-AN who were working at a specialist child and adolescent eating disorder service responded to an online survey about their experience of including a dietitian in FT-AN and how they understand the role. Both categorical and open-ended questions were used. Reflexive thematic analysis was used to analyse the qualitative free-text responses of clinician perspectives on the role of the dietitian in FT-AN. Results: All clinicians agreed that dietetics had a role within FT-AN and most frequently sought dietetic involvement in the early phases of FT-AN. Reflexive thematic analysis of responses identified three main themes. These were (1) collaboration is key, (2) confidence as a core consideration and (3) case-by-case approach. These themes evidenced the role of the dietitian within FT-AN and highlighted both the benefits and concerns of this involvement. Conclusions: This study demonstrated that dietitians can take a core role as collaborators within therapy-led teams that facilitate joint working and sharing of expertise. However, dietetic input should be considered on a case-by-case basis, given its potential for creating an over-focus on nutrition and potentially diminishing parental confidence in feeding. When indicated for selected cases, nutritional counselling should be offered in joint sessions with the therapist rather than separately. The findings of the study were limited by the small sample size of participants recruited from a single centre and heterogeneity in the professional background of respondents. Although the integration of dietetics within the multidisciplinary team and the ability of dietitians to individualise patient care can enhance FT-AN treatment, potential benefits and disbenefits should be considered for each case.

## 1. Introduction

Anorexia nervosa (AN) is a psychiatric disorder, affecting both physical and mental health. It is the third most common chronic illness among adolescent females and has one of the highest mortality rates amongst all psychiatric conditions [1], ranking second after substance abuse [2]. AN is associated with severe medical, nutritional and psychological consequences and the treatment necessitates input from professionals with specialist skills to manage each of these domains [3,4,5]. In treatment models for adolescent AN, the family is seen as a valuable resource in supporting the recovery of the young person from the illness [6,7]. As such, interventions employing a family-based approach for treating AN in young people are recommended by the national guidance from Australia and New Zealand [8], the Netherlands [9], Spain [10], the United Kingdom (UK) [11], the United States of America (USA) [12], Denmark [13] and France [14]. 

Family therapy for anorexia nervosa (FT-AN) is recommended as a first-line treatment for young people with AN by the National Institute for Health and Care Excellence (NICE) [11]. A multidisciplinary team (MDT) approach is described as paramount in both the FT-AN manual [15] and the current UK guidance and standards on specialist child and adolescent eating disorder service structure [16] to ensure that holistic care (including, nutritional, physical monitoring, medication and psychiatric assessment) is offered. Dietitians, alongside other key professions, are described as core members of this MDT [15,16]. 

Dietitians are skilled in the assessment and management of disordered eating patterns, malnutrition, underweight and nutritional-related deficiencies [17,18]. Restrictive eating, low weight and malnutrition are all salient characteristics of AN [19]. Dietitians are well-placed to support nutritional-related issues arising from the illness. However, eating disorder-associated behaviours and their impact on nutrition are symptoms of the illness, rather than the cause. Dietetic interventions, aimed at managing malnutrition and supporting refeeding, are often recommended as part of the specialist MDT, whereby psychological therapy (such as FT-AN) and medical management are central parts of treatment that are delivered by appropriately qualified and experienced professionals [20,21]. 

Dietitians are routinely involved in inpatient, residential and day treatments across the life span in AN. However, the role of dietitians across outpatient treatments for children and adolescents with AN is highly variable. Within family approaches to the treatment of AN, there has been some difference in opinion over whether or how a dietitian should be involved. For example, the family-based treatment (FBT) manual does not recommend dietitians as part of the direct treating team; however, dietitians are mentioned as potential members of the consulting team, and in clinical practice, dietitians routinely work in parallel with FBT therapists [22,23,24]. In FBT, the nutritional guidance on weight restoration delivered by a dietitian is perceived to have the potential to undermine parental confidence in their ability to feed their child and make healthy food choices for them, just as they had done prior to them becoming unwell with AN. In the Maudsley FT-AN manual, however, dietitians extend the nutritional knowledge base of the MDT, providing nutritional psychoeducation in collaboration with therapists and devising standard meal plans that are used to provide guidance in the initial stages of the treatment [15]. In FT-AN, it is suggested that dietitians can also take a more direct advisory role when needed at several stages across treatment. This can include case consultation if weight restoration is not progressing, modifying standard meal plans when there are specific dietary requirements (e.g., coeliac disease) or supporting the young person to move to independent eating. Joint working between the dietitian and therapist is recommended to take place in family sessions to ensure MDT collaboration with the family. Given these conflicting views about how and when to involve dietetics within two very similar treatment models, further research exploring dietetic roles in the outpatient treatment of eating disorders in children and adolescents is needed, in addition to clear guidelines describing the role of the dietitian in FT-AN [18]. 

Existing research studies mainly concentrate on the role of the dietitian in adult eating disorder treatment and highlight dietitians as essential in the management of malnutrition, whilst also needing to work collaboratively and in accordance with psychological and medical aspects of the treatment [18]. Despite dietitians being skilled in assessing and treating malnutrition, a core characteristic of eating disorders, their involvement in treatment has been raised as a contentious issue [25]. A study by McMaster et al. employed a modified Delphi method to develop consensus guidelines across multiple domains of dietetic involvement within eating disorder treatment for adults. Results identified disagreement between clients and clinicians on essential components of dietetic treatment. Dietetic input was more highly valued by clients than clinicians, and this was raised as a potential barrier to the involvement of dietitians in the treatment, given that access to dietitians is often reliant on referrals being initiated by therapists [25]. 

Research and guidance on the dietitian’s role in adolescent eating disorder treatment are lacking. No empirical studies have been conducted, to date, which assess the effect of nutrition counselling on treatment outcomes in FT-AN or FBT. In the absence of such studies, the ability to advance our understanding of the role of dietitians in treatment remains limited. Though the roles and responsibilities of the dietitian have been conceptualised in FT-AN treatment, further guidance on the role of the dietitian within FT-AN is required to support best practices in this area and ensure consistent evidence-based practice across services delivering this treatment. This study aimed to gather perspectives from experienced clinicians, specialising in the delivery of FT-AN, on the role of the dietitian in FT-AN. The objective of the study was to take an initial step towards gathering evidence that supports further research in this area and will aid the development of consensus guidelines on this role. 

## 2. Materials and Methods

### 2.1. Sample 

All clinicians who were working at the Maudsley Centre for Child and Adolescent Eating Disorders (MCCAED) outpatient service and had experience delivering FT-AN during the data collection period were invited to participate. All clinicians were qualified family therapists, psychiatrists, nurse therapists or clinical psychologists with training and experience in FT-AN. MCCAED provides specialist treatments for all children and adolescents with eating disorders for an area of Southeast London with a population of approximately 2.2 million people. The outpatient service provides specialist family and psychological therapies, psychiatric management, dietetics and physical health reviews. The primary treatment model for anorexia nervosa is FT-AN (see recent publications for service details and outcomes [26,27]).

### 2.2. Procedure

Approval for this project was granted by South London and Maudsley Child and Adolescent Mental Health Services (CAMHS) service evaluation and audit committee (approval number 330 and date 23 August 2023). Surveys were used to collect data from clinicians via an online platform [28]. Survey questions were informed by MDT consultation about core components of dietetic treatment within FT-AN, barriers to dietetic treatment and outcomes related to dietetic involvement in FT-AN. Surveys were used to allow participants to respond anonymously and remove any potential sources of bias in their responses. Data were collected between June and July 2023. The survey was sent to all clinicians (*n* = 35) with experience in delivering FT-AN and working within the MCCAED team. Surveys recorded the profession of the respondent and were made up of 10 questions allowing quantitative rating and categorical responses. This was followed by 7 open-ended questions, which explored opinions on the role of dietetics in FT-AN, the effects of including a dietitian in FT-AN and potential benefits and challenges that arise when directly or indirectly involving dietetics as part of FT-AN. 

### 2.3. Analysis Plan

Quantitative data from the survey questions are used descriptively rather than to test statistical hypotheses. Qualitative data were separately analysed using reflexive thematic analysis within a critical realist framework, which views meaning and experience as subjective and influenced by social and cultural context. Responses and comments on open-ended questions were initially coded, and initial themes were generated. The themes were reviewed and developed before being defined. Themes were developed through reflexive engagement with the data, with the involvement of two authors who were both working in the service at this time (CB and JB). Themes were cross-checked with survey respondents, and comments or feedback were used to adapt the themes to accurately reflect the views of the team. 

### 2.4. Reflexivity Statement 

CB is a cisgender white female working as a dietitian within the FT-AN model in MCCAED, she understands and is aware of the roles and responsibilities that surround being a dietitian in this team and delivering this treatment model. The questions asked within the survey and the themes drawn from responses will inherently contain biases due to their role in the team. The results and conclusions come from their perspective, and their awareness of these biases has been taken into account. 

JB is a cisgender white male working as a clinical psychologist within the FT-AN model at MCCAED. He has more than 10 years of experience clinically delivering family-based treatments for adolescent AN, as well as teaching and conducting research in the area. He has provided treatment within both the FBT and FT-AN models and has experience of performing this with and without dietetic input. This experience will inevitably bring bias to the data analysis, although having had the experience of both types of family treatments and performing so with and without a dietitian brings depth to the analysis. 

## 3. Results

### 3.1. Sample

Twenty clinicians responded to the survey, and these were from a range of professional backgrounds, including nurses (*n* = 1), psychiatrists (*n* = 1), psychologists (*n* = 6) and family therapists (*n* = 5), who represented the MDT. Seven respondents did not disclose their professional background. 

### 3.2. Quantitative Data

Descriptive data for responses to categorical survey questions are displayed in Table 1 for all survey respondents (*n* = 20). All clinicians included in the survey had experience working with a dietitian when delivering FT-AN. Dietetic input was sought for differing proportions of clinicians’ caseloads and most frequently for less than 10% (*n* = 6) and up to 25% (*n* = 7) of FT-AN caseloads. All clinicians agreed that dietetics had a role within FT-AN (*n* = 20) and that they most frequently sought involvement in phases 1 and 2 of FT-AN. 

Opinions were divided on the potential for dietetic involvement in FT-AN to be unhelpful, with 45% of clinicians (*n* = 9) stating that there could be unhelpful aspects, whilst 55% (*n* = 11) did not think there were aspects that could be unhelpful. The majority of clinicians agreed that the therapeutic alliance was affected by involving dietetics within FT-AN (65%, *n* = 13). Most clinicians also agreed that there was a greater or differing need for dietetic involvement in atypical cases during FT-AN (65%, *n* = 13). 

All clinicians reported that if standardised meal plans were provided, these were used within phases 1 and 2 of FT-AN, with no clinicians reporting their use in phase 3 or 4 (75%, *n* = 15 and 30%, *n* = 5, respectively) All clinicians agreed that there were indirect benefits of having a dietitian as part of the MDT when delivering FT-AN (100%, *n* = 20). 

### 3.3. Qualitative Data

Analysis of free-text responses generated three main themes and eight subthemes that applied to all clinicians (see Table 2). The themes were (1) collaboration is key, (2) confidence as a core consideration and (3) a case-by-case approach. Sub-themes were (1a) working together, (1b) learning from each other and (1c) building trust; (2a) timing matters and (2b) skilling up; (3a) assessing needs, (3b) individualised treatment and (3c) moving on from meal plans. Each one is described further below with relevant illustrative quotations.

1. Collaboration is key.

1a. Working together. A core theme that was evident amongst all clinicians related to collaborative working between dietitians and therapists and professionals and families. Responses reinforced fidelity to the FT-AN approach, whereby all MDT members provided specialist input as a cohesive joined-up team. Incorporating dietetics within the treatment rather than as an isolated or separate intervention was important to clinicians. Concerns were raised regarding team splitting and diminished therapeutic alliance when dietetic involvement was offered as a separate intervention.

*“I think it is unhelpful if this is done separately and not as part of sessions/treatment. If it is separated it can impact engagement, the therapeutic relationship and learning from each other.”*

*“Most importantly though their role is to be a collaborative partner with the therapist and family against the illness, rather than being a separate voice/perspective.”*

*“Our treatment model is based on delivering treatment within an expert MDT that involves the combined knowledge of all disciplines. Phase one of treatment is based on engaging the family with the MDT approach and creating a secure base for treatment”* *… “So, all of the above but together with care coordinator/therapist and based on formulation of need to do something different rather than totalistic approaches.”*

1b. Learning from each other. Sharing of knowledge and expertise was another core component of dietetic involvement in FT-AN. Clinicians frequently reported that their confidence and knowledge regarding nutritional aspects of treatment were greatly increased by working with a dietitian, which enhanced their ability to deliver FT-AN. The consensus was that this transfer of information and learning from colleagues was bi-directional, whereby therapists and families benefited from dietetic expertise, and similarly, dietitians expanded and improved their practice through learning from therapists and families.

*“I have learned a lot working together with a dietitian over the years and it has contributed to my development and expertise, and I hope this was bidirectional.”*

*“Working together is essential to safe practice and there is a lot of learning that happens both ways across disciplines.”*

1c. Building trust. Creating a stable base for FT-AN is essential in the delivery of this treatment. Clinicians felt that collaboration as an MDT, working together and supporting families jointly in psychological and nutritional aspects of treatment were important in building trust and engagement. The consensus from clinicians was that the involvement of a dietitian, as a planned intervention, supported the therapeutic alliance and strengthened families’ trust in the treatment. 

Bringing in an expert on nutrition in certain cases during FT-AN-aided containment at different phases of treatment, depending on the family’s needs. Concerns were raised by clinicians regarding the involvement of a dietitian when a strong therapeutic alliance with the family had not yet been built, or when engagement was already poor. The involvement of dietetics in these scenarios could have a negative impact in terms of undermining the therapist or parents or creating an overreliance on the dietitian in meal planning.

*“I think there are a few ways the dietitian is really helpful”* *… “in containing anxiety about families feeling like they are doing the ‘right thing’, offering support and comfort to the clinician that things are on the right track (more of a distant role) and in providing the same message as the therapist but from a different perspective (e.g., united team front).”*

*“I think if families are motivated and on board to see a dietician then it works better. Sometimes it can feel unhelpful when the parents have pre-existing beliefs about a dietician being able to “solve” the young person’s eating problems instead of parents needing to take an active role in FT AN.”*

2. Confidence as a core consideration.

2a. Timing Matters. All clinicians agreed that the timing of dietetic involvement was an important consideration. Parental confidence could be negatively impacted by dietetic intervention too early in treatment, prior to a strong therapeutic alliance being built. Similarly, clinicians voiced concerns regarding dietetic involvement inadvertently undermining parental confidence in meal planning, portioning and feeding if the input was poorly timed.

*“I think if the family is motivated and on board to see a dietician then it works better.”*

*“Providing expert consultation if the family is really lacking knowledge and confidence in what types of foods to give, in containing anxiety about families feeling like they are doing the ‘right thing’, offering support and comfort to the clinician that things are on the right track (more of a distant role) and providing the same message as the therapist but from a different perspective (e.g., united team front).”*

2b. Skilling up. Survey respondents made multiple references related to the use of dietetic sessions and input to support both clinician and parental confidence in several nutritional aspects of the treatment during FT-AN. There was an overall consensus that dietitians had a central role in sharing expertise on nutritional information, meal planning and intuitive eating with families and in supporting them to feel confident in taking responsibility for these aspects of the treatment. The importance of a collaborative, joint-up approach was reiterated by clinicians. If the approach was split or not collaborative, clinicians felt that the confidence of both clinicians and parents could be negatively impacted.

*“I think it can also affect clinical confidence if not done jointly.”*

*“Helping give confidence and containment to families taking positive risks to have meals not guided by the meal plan which are more in line with “real life”.”*

3. Case-by-case approach.

3a. Assessment of needs. Dietetic involvement was considered an important and valuable element of treatment within the FT-AN model. However, clinicians felt that an assessment of the needs of the family was crucial before seeking dietetic input. Families requiring dietetic involvement for core purposes such as refeeding management, meal planning, special or complex diets and increasing variety with food were referenced as appropriate uses of dietetic input. In the absence of a clear need, dietetic involvement was seen as unnecessary. Where dietetic involvement was not indicated, clinicians were concerned that the input could lead to an over-focus on nutrition and the potential avoidance of therapeutic work.

*“What can also be unhelpful is to take extreme views (i.e., everyone needs to be seen by dietician) as it does not acknowledge the existing processes in place and also other ways that expertise can be shared.”*

*“Sometimes it can feel unhelpful when the parents have pre-existing beliefs about a dietician being able to “solve” the young person’s eating problems instead of parents needing to take an active role in FT AN.”*

*“Sometimes the families think that if they ‘only got the food right’ then the eating disorder would be fixed. Given it is part food part emotion, if we over-focus on the food, I think this can act like an avoidance of the actual issue.”*

3b. Individualised treatment. Another important role of the dietitian within FT-AN discussed by clinicians was individualising feeding plans, in particular, in adapting meal plans or nutrition advice for bespoke plans and advice. Clinicians felt that this supported a patient-centred approach and helped to ensure that individual needs could be met. Cases with specific dietary needs, complex physical health co-morbidities, atypical presentations or cultural diversity were seen to benefit from direct dietetic input.

*“I think it would be very helpful to have more dietetic role in FT-AN especially when working with young people who are not underweight and also binge/purge.”*

*“Would also want dietetic support (in atypical cases) so not promoting any anti-fat messages unintentionally, especially if someone’s weight is at higher end.”*

3c. Moving on from meal plans. Meal plans were discussed by all clinicians, and there was a general consensus that, despite them supporting containment and providing guidance early on in treatment, they could often become unhelpful when families remain “stuck” on them. Clinicians felt that dietitians had a key role in helping families move away from meal plans at various points through FT-AN. During the initial phases, direct or indirect dietetic consultation was used as a tool to educate and guide on refeeding in the absence of a meal plan. In later phases of treatment, the dietitian’s role was related to moving off the initially prescribed meal plan and/or incorporating greater dietary variety and flexibility than that provided on standardized meal plans. Again, the theme of building confidence in the nutritional aspects of treatment and working collaboratively to achieve goals was referenced by clinicians.

*“When starting to have more independence and eating on their own, going out more, dietetic input would be very helpful. Also, when parents or YP (young people) feel worried to move away from meal plan.”*

*“Planning with the dietitian can make the young person feel safe and confident to take on responsibility for their eating.”*

*“Other times I think older young people need support in understanding the truth about nutrition to help them make better choices, e.g., where social media and googling has provided unhelpful advice.”*

## 4. Discussion

Currently, no empirical studies exist that investigate the role of dietitians in family treatments for anorexia nervosa. This study provided initial insights into this topic. 

In this study, the exploration of MDT perspectives on the role of the dietitian within FT-AN supported dietetic involvement in some cases. Dietitians were seen by experienced clinicians as a valuable resource, which should be considered and offered when indicated for selected families. Access to dietetic expertise was most valued during the initial phases of the treatment, where nutritional rehabilitation most often takes place, and in atypical cases presenting at healthy weights or for cases with co-morbid complexities. Having said that, direct input from a dietitian was not considered necessary for all and needed to be considered on a case-by-case basis. The involvement of dietetics, where no clinical need was identified, was considered by nearly half of clinicians surveyed to be potentially unhelpful. The findings of this study highlighted several barriers that may discourage therapists from seeking dietetic involvement, including concerns related to diminishing parental confidence in feeding and creating an over-focus on the nutritional aspects of the treatment. 

A recent review by Heafala et al. explored the role of dietitians in eating disorder treatment [29], including perspectives of dietitians, MDT members and service users. The themes that were generated from the review were similar to those identified in the current study. The role of dietitians was varied, encompassing roles as collaborators, educators and in supporting individualised patient-centred care [29]. Concerns regarding the impact of dietetic involvement on certain aspects of care that were identified included uncertainty regarding the scope of practice and unclear treatment guidance [29]. McMaster et al. also found that there were a number of barriers to dietetic involvement in eating disorder treatment related to the perception that inexperienced dietitians may discuss weight loss or dieting with patients [30]. The need for clarity on the role of the dietitian, criteria necessitating dietetic involvement and clear guidance on the structure of dietetic involvement within eating disorders has been repeatedly highlighted [29,31,32]. Future directions should include a range of appropriately designed and well-powered empirical studies on this subject to provide much-needed, concrete evidence on the impact that dietitians may have in treatment. 

### Strengths and Limitations 

A strength of this study was in its novel nature, namely that it is the first study that provides an initial important step analysing descriptive, as well as qualitative data on the involvement of dietitians in outpatient FT-AN. Other strengths include its setting, a highly specialised eating disorder centre, where FT-AN was conceptualised, developed and the range of professions included within the sample that spanned multiple disciplines. 

The study had a number of limitations. The findings from a convenience sample of self-selecting clinicians from a single centre may not generalise to all clinicians delivering comparable treatments in similar settings. Clinicians who responded may have had more positive views of dietetics, introducing potential biases. However, the use of anonymous surveys aimed to reduce potential biases and support clinicians to answer questions truthfully, including both positive and negative responses. Similarly, although all clinicians surveyed were working primarily as therapists delivering manualised FT-AN, respondents did span a range of professional backgrounds. Experiences and perceptions of the role of the dietitian in FT-AN may have been impacted by professional background. Additionally, this was a single-centre study, meaning that results may not be representative of other eating disorder centres, where experiences and perceptions of the dietitians’ role may differ.

## 5. Conclusions

Although manualised FT-AN clearly describes the dietitian as a core member of the treating team, the roles and responsibilities of dietitians in eating disorders-focused family therapy remain a contentious issue. The findings of this study highlight collaboration as a key component of the dietitian role. To enable the creation of dietetic treatment guidelines that include the MDT perspectives, collaboration with clinicians delivering FT-AN was seen as an important first step. To further advance our understanding of the topic, high-quality research studies are needed. Previously proposed guidance on the dietitians’ role in FT-AN originated predominantly from dietitians [33], potentially lacking the diverse perspectives of other professions involved in delivering FT-AN, and the robust research methods required to provide high-quality evidence needed for the development of such guidance. Further research is essential in this area to provide sound evidence on the role of dietitians within family-based treatments for anorexia nervosa. 

The overall consensus from clinicians surveyed in our study provided guidance on three important components for dietetic involvement, and further research, in FT-AN. We propose the following treatment implications related to these core components of the role of dietitian within FT-AN. 

## 6. Recommendations for Clinical Practice

1. Dietitians should be recognised as collaborators within therapy-led teams. There should be a focus on joint (family–therapist–dietitian) working that facilitates discussions between therapists and dietitians, working together with parents and families and enhancing the therapeutic alliance through supporting individual patients’ goals through collaboration between dietitians, therapists and families.
2. Dietitians should be integrated within the MDT and have direct and indirect involvement in patient care. Dietitians have an important role that involves liaising with dietitians and staff from external teams. Indirect dietetic involvement should include resource creation and professional consultation. Direct involvement should be offered when clinically indicated when co-morbid physical health problems exist, in cases of complex dietary needs, such as allergies, intolerances or sensory sensitivity, and in cases where nutritional requirements are difficult to meet through food alone and supplementation or tube feeding may be required. Dietetic involvement should support increased confidence in the MDT and the sharing of skills and nutritional expertise. Training between dietitians and teams should be bidirectional with knowledge sharing and training occurring from therapist to dietitian and vice versa to support all staff in “singing from the same hymn sheet”.
3. Dietitians have a core role in individualising care and ensuring that the diverse needs of families being treated by the service are met. This involves bespoke meal planning when required or providing tailored nutritional recommendations for families with individual needs with co-morbid illnesses, individuals who are highly active or athletic and those who present at higher weights that require additional nutritional advice within FT-AN.

## Figures and Tables

**Table 1:** Quantitative survey response data from participants (*n* = 20).

Survey Question	*N*	%
What percentage of your caseload have you sought dietetic input for?		
0%	1	5
<10%	6	30
10–25%	7	35
25–50%	2	10
50–75%	1	5
<75%	3	15
Within what phase of FT-AN is dietetic input most valuable?		
No phases	0	0
Phase 1	6	30
Phase 2	6	30
Phase 3	0	0
Phase 4	3	15
All phases	5	25
What phase of treatment would you typically use the standard meal plans?		
Phase 1	15	75
Phase 2	5	30
Phase 3	0	0
Phase 4	0	0

**Table 2:** Themes and subthemes of reflexive thematic analysis.

Themes	Sub-Themes	Codes
Collaboration is key	Working together	1a
	Learning from each other	1b
	Building trust	1c
Confidence as a core consideration	Timing matters	2a
	Skilling up	2b
Case-by-case approach	Assessing needs	3a
	Individualised treatment	3b
	Moving on from meal plans	3c

## Data Availability

Data is available on request. Please contact the corresponding author for data requests.
